# Radiographic and cone-beam computed tomography characterization of Chevron lucency in 82 clinically healthy maxillary canine teeth of dogs: a retrospective descriptive study

**DOI:** 10.3389/fvets.2026.1876288

**Published:** 2026-07-01

**Authors:** Lenin A. Villamizar-Martinez, Yang Qu, Cristian M. Villegas Lobos, Fernanda M. Lopes-Kubitza

**Affiliations:** 1 Dentistry and Oral Surgery Service, Department of Clinical Science, North Carolina State University, Raleigh, NC, United States; 2 Clinical Studies Core, Office of Research, College of Veterinary Medicine, North Carolina State University, Raleigh, NC, United States; 3 Luiz de Queiroz College of Agriculture, São Paulo University, Piracicaba, SP, Brazil

**Keywords:** Chevron lucency, cone beam tomography, diagnostic imaging, dogs, intraoral radiography, periapical lucency, tooth apex

## Abstract

The apex of the canine tooth is surrounded by the periodontal ligament and alveolar bone, where the periapical cancellous bone may radiographically present as a V-shaped radiolucent area known as the “chevron lucency” (CL). Although CL in healthy maxillary canine teeth of dogs is considered a normal radiographic finding, its characterization on dental radiographs remains challenging due to the two-dimensional limitations of this imaging modality. This retrospective descriptive study aimed to morphologically characterize the radiographic and cone-beam computed tomography (CBCT) features of CLs in clinically healthy maxillary canine teeth of dogs and to determine which imaging modality provided superior diagnostic reliability for their identification and classification. Dental radiographs and CBCT images of 82 clinically healthy maxillary canine teeth from 41 dogs were retrospectively analyzed to evaluate key imaging features of the periapical region, including CL presence, shape, symmetry, lamina dura appearance, periodontal ligament space, and periapical cancellous bone pattern. CBCT demonstrated almost perfect inter-observer agreement for CL presence, shape, and symmetry (*κ* > 0.81), outperforming intraoral radiography, which showed only moderate-to-substantial agreement for the same features (*κ* = 0.46–0.78). The irregular shape was the most common CL morphology in both imaging modalities (47.7% in radiography; 65.7% in CBCT). Body weight and sex were the primary predictors of CL identification, with heavier dogs showing higher odds of CL detection (OR = 1.17 per kg; 95% CI: 1.06–1.28). In conclusion, CBCT provides greater diagnostic reliability than intraoral radiography for characterizing CL and periapical structures in maxillary canine teeth of dogs and should be considered when radiographic findings are inconclusive or detailed morphological assessment is required for clinical decision-making.

## Introduction

The alveolar process of the incisive, maxillary, and mandibular bones forms the dental alveoli, the cavities that house the deciduous and permanent teeth in the dog (1). The alveolar process comprises an external cortical plate formed by Haversian compact bone, cancellous bone, and an inner, thinner compact bone lamina filled with microscopic perforations named the cribriform plate. This cribriform plate is the portion of the dental alveolus that allows the passage of the neurovascular supply from the alveolar bone to the periodontal ligament and cellular portion of the cementum at the tooth root (2).

Radiographically, the alveolar bone is typically characterized by a radiopaque, homogeneous trabecular pattern. It is lined at the alveolar margin and within the alveolus by a radiopaque linear structure corresponding to the cortical bone. The radiopaque linear image along the inner wall of the alveolus is termed the “*lamina dura”* and anatomically corresponds to the *“cribriform plate.”* The periodontal ligament (PDL) appears as a parallel radiolucent linear space adjacent to the lamina dura around the root. Depending on the radiographic appearance, the apical space may appear enlarged in healthy teeth and is sometimes referred to as a chevron lucency or sign (3, 4).

Therefore, a chevron lucency (CL) is a V-shaped periapical radiolucency associated with the normal trabecular alveolar bone pattern. It is most seen at the incisors and canines, but it may be present apical to any tooth (3, 5). In dogs, it has been suggested that the CL may provide additional cushioning at the apical region of the teeth due to thinner trabecular bone, marrow spaces, and vascular channels that may absorb and dissipate stress during biting and mastication. However, biomechanical studies confirming such a function are currently lacking.

A periapical lesion (PAL) may develop when pulp infection extends to the periapical area through the apical canal or delta. This can lead to clinical changes such as swelling and a draining tract, depending on the patient’s immune status, the injury’s acuteness or chronicity, and the virulence of the insult (6–8). Differentiating a CL from an actual PAL can be challenging on two-dimensional intraoral radiographs, as both may show similar radiographic features. In acute stages, a PAL may show widening of the periapical ligament space and loss or disruption of the lamina dura, progressing to a more extensive periapical radiolucent lesion. In chronic stages, radiographic features may include apical tooth resorption, sclerosis of the periapical alveolar bone, periosteal reaction of the adjacent cortical bone, and bone expansion (6, 9). In contrast, the radiolucency of a CL corresponds to normal alveolar bone conformation.

Although advanced multiplanar diagnostic techniques, such as computed tomography (CT), magnetic resonance imaging (MRI), and cone-beam computed tomography (CBCT), have demonstrated their superiority over bidimensional techniques like intraoral radiographs in human and veterinary dentistry for assessing the anatomy and status of endodontically diseased or treated teeth (10–13), no studies have been conducted in veterinary patients to specifically assess the periapical region of clinically healthy teeth using CBCT. Thus, the objective of the study reported here was to morphologically characterize the radiographic and cone-beam computed tomography (CBCT) features of CLs in clinically healthy maxillary canine teeth of dogs, and to determine which imaging modality provided superior diagnostic reliability for their identification and classification.

## Hypothesis

Our null hypothesis was that both intraoral radiography and CBCT could identify the CL, and our alternative hypothesis was that CBCT, by eliminating structural superimposition and enabling multiplanar visualization, would outperform intraoral radiography in characterizing the morphology of CL and related periapical structures.

## Materials and methods

### Study design and sample selection

Clinical records, intraoral radiographs, and CBCT images of maxillary canine teeth from client-owned dogs with different skull conformations, obtained between 2021 and 2025 at the Dentistry & Oral Surgery Service, College of Veterinary Medicine of North Carolina State University, were retrospectively evaluated by two board-certified veterinary dentists (Diplomates of the American Veterinary Dental College). The study focused on 82 clinically healthy maxillary canines from 41 dogs of various skull conformations and breeds, sexes, and ages. Only patients with intraoral radiographs of one or both maxillary canine teeth and CBCT imaging of the maxillary canine region were included. For skull conformation, dogs were classified by skull index from CBCT multiplanar reconstructions (1).

Cases that, on physical examination, showed evidence of a neoplastic process in the rostral maxillary area, or maxillary canine teeth with discoloration, dental fracture, dentoalveolar trauma, or that, on dental imaging (radiographic or CBCT), showed signs of severe periodontitis or endodontic disease, were excluded from the study. Imaging features associated with severe periodontitis and endodontic disease include horizontal or vertical bone loss affecting the apical third of the root, alveolar sclerosis, alveolar bone expansion, periosteal reaction adjacent to the cortical bone near the apex of the evaluated tooth, widening of the pulp cavity compared with the homologous contralateral tooth, internal or external inflammatory tooth resorption and the presence of PAL.

This retrospective study was based exclusively on existing clinical records and diagnostic images collected during routine veterinary care at the Dentistry and Oral Surgery Service, North Carolina State University College of Veterinary Medicine. No experimental procedures were performed, and IACUC approval was not required. Client consent for the use of anonymized data for research purposes was obtained in accordance with institutional policy.

### Radiographic and CBCT imaging

Eighty-two lateral intraoral radiographs and 82 CBCT images of the right and left maxillary canine teeth, originally obtained from patients presented for professional dental cleaning and periodontal therapy, were retrospectively reviewed by two observers, resulting in 164 evaluations (82 teeth × 2 evaluators). All imaging was performed by two veterinary dental technicians, one resident, and/or two board-certified veterinary dentists. Intraoral radiographs were acquired using a standardized technique at 60 kV and 2.5 mA with patients in sternal or lateral recumbency, while CBCT images were obtained using a mobile unit with the patient’s head in ventral recumbency. The CBCT scans followed a protocol with a voxel size of 0.3 mm, a 24 × 14 cm field of view, 120 kVp, 57.6 mAs, and a scan duration of 20 s.

**Figure 1:**
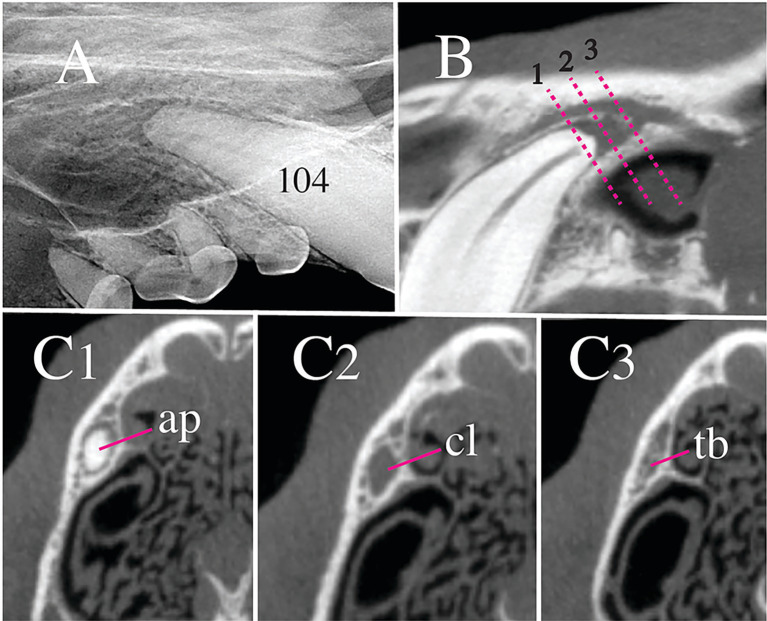
Example imaging set of the maxillary right canine tooth (104) used to evaluate the apical and periapical anatomical features of a six-year-old Golden Retriever: **(A)** dental radiograph, **(B)** CBCT sagittal, and **(C1-3)** transverse reconstructions. (B) Dashed lines in the sagittal reconstructions indicate the anatomical regions where the transverse reconstructions were performed: (**1-C1, 2-C2, 3-C3)**. Tooth apex (ap), chevron lucency (cl), and trabecular bone distal to the chevron lucency (tb).

Both the radiographs and CBCT scans were initially saved in DICOM (Horos, version 3 (LGPL-3.0) format and assigned identification numbers to prevent correlation between the two modalities during subsequent evaluation. For this study, one author converted these datasets into standardized JPEG image packages. For each dog, the intraoral radiography packages consisted of two images (one for the right and one for the left maxillary canine), while the CBCT packages included one sagittal and three transverse reconstructions of the apical and periapical areas of each tooth. These CBCT images were specifically processed using a suitable bone level and window to optimize diagnostic quality. Figure 1 presents the imaging set and the regions used to perform the apical and periapical anatomical evaluations.

To facilitate a blind assessment, all images were adjusted for standardized brightness and contrast and stripped of all patient demographic information. Prior to evaluation, reviewers participated in a training session to standardize the identification of anatomical features and the application of the scoring system. The blinded evaluation using the proposed scoring was conducted on a 4.5 K monitor (2024 24-inch iMac Retina display). During the assessment, the intraoral radiographs were presented and graded in a single initial session. One week later, the corresponding CBCT images were released and reviewed in a separate single session.

### Anatomic evaluation

**Figure 2:**
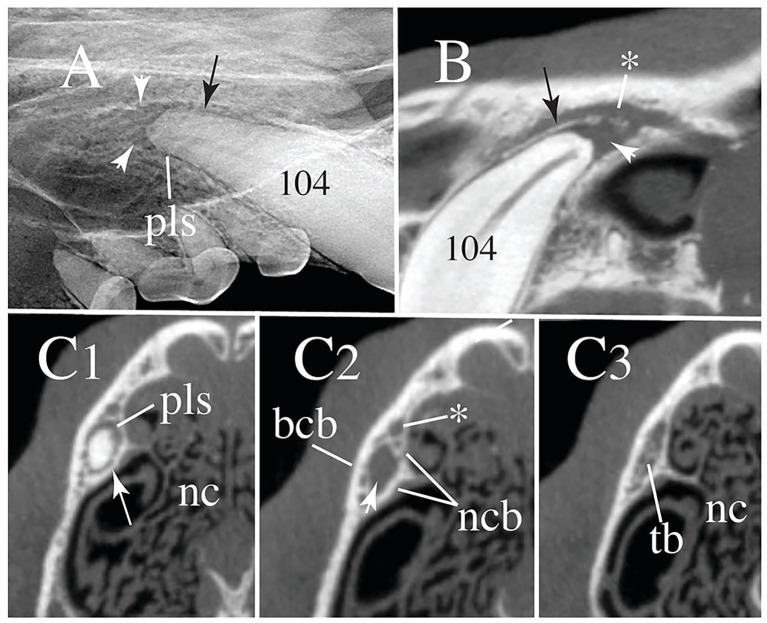
**(A)** Dental radiograph, **(B)** CBCT sagittal, and **(C1–C3)** transverse multiplanar reconstructions of the maxillary right canine tooth (104) of the dog presented in Figure 1, depicting the apical and periapical anatomical features evaluated in this study: **(A,B,C1)**: chevron lucency (arrowheads), lamina dura (arrow), and periodontal ligament space (pls); **(C2)**: buccal and nasal cortical bone adjacent to the apex (bcb–ncb); and **(C3)**: cancellous or trabecular bone (tb). Note that a fine cortical bone lamina corresponding to the lamina dura at the apex separates the chevron lucency from the incisivomaxillary canal (asterisk) in **(B)** and **(C2)**. (nc) nasal cavity.

Two reviewers, blinded to each other, independently assessed six anatomical features in both intraoral radiographs and CBCT images (Figure 2): presence of CL, CL shape, left–right maxillary canine CL symmetry, lamina dura appearance, periodontal ligament space appearance, and periapical cancellous bone pattern. A seventh feature, the cortical bone adjacent to the apex (facing the nasal cavity and the buccal plate), was evaluated solely on CBCT images. The variables assessed and their corresponding scoring criteria are summarized in Table 1.

**Table 1:** Evaluation criteria for periapical features and chevron lucency (CL) in maxillary canine teeth using intraoral radiography (IR) and cone beam computed tomography (CBCT).

Morphologic feature	Scores for IR and CBCT
CL Presence	(0) Not visible, (1) partially seen or poorly defined, (2) well defined, ‘V’ shape or other shape
CL Shape	(0) ‘V’ shape, (1) round, (2) irregular, (3) absent
CL Symmetry	(0) Symmetrical, (1) asymmetrical
Lamina Dura	(0) Not visible, (1) partially seen or poorly defined, (2) well defined radiopaque continuous cortical line
PDL Space	(0) Not visible, (1) partially seen or poorly defined, (2) continuous radiolucent/hypoattenuating line around the tooth root apex
Cancellous Bone Pattern	(0) Not visible, (1) partially seen, poorly defined, (2) fine, uniform trabecular bone
Cortical bone near apex *	(0) Not visible, (1) partially seen, poorly defined, (2) well defined, continuous, uniform maxillary, nasal and labial cortical bone

PDL, Periodontal ligament; * Only evaluated in CBCT images.

### Data analysis

Data analysis was performed in R 4.5.3 (R Core Team). Demographic data, including age, body weight, sex, and skull conformation, were summarized for 41 animals, and score distributions for CL shape were described across 82 observations (2 teeth × 41 animals). Three main analyses were conducted on the 82 observations: (1) Inter-observer reliability for both IR and CBCT was assessed using weighted kappa (*κ*) with quadratic weighting (kappa2 function, irr package) (14), interpreted according to the Landis and Koch scale; (2) Radiographic diagnostic performance relative to CBCT (treated as reference standard) was evaluated per score level using a one-vs-all approach, reporting sensitivity, specificity, positive predictive value (PPV), negative predictive value (NPV); (3) Association between CBCT CL presence and covariates (sex, age, body weight, and skull conformation) was explored using cumulative link mixed models (clmm) function, ordinal package (15), with model assumptions verified and odds ratios reported for significant predictors. A significance level of 0.05 was applied throughout.

## Results

Eighty-two maxillary canine teeth from 41 dogs met the inclusion criteria for this study. Among the study population, 24/41 (58.5%) were male and 17/41 (41.5%) were female. Dogs with mesaticephalic skull conformation were the most prevalent, with 35/41 (85.4%) of the population. This was followed by dogs with dolichocephalic (4/41, 9.8%) and brachycephalic (2/41, 4.8%) skull conformations, respectively. The demographic data on age and weight are presented in Table 2.

**Table 2:** Demographic distribution (age and weight) of the study sample (*n* = 41).

Variable	Mean	SD	Median	Q1	Q3	Range (Min–Max)
Age (Months)	95.7	40.6	90.0	63	120	18.0–175.0
Weight (kg)	17.1	12.2	12.2	5.7	29.1	2.63–41.5

kg, Kilograms; Q1, First Quartile; Q3, Third Quartile.

Statistical analysis showed that CBCT imaging provides higher diagnostic reliability than intraoral radiography for evaluating CL. Inter-observer agreement for CBCT was almost perfect for CL symmetry, CL shape, and CL presence, with kappa values > 0.81. In contrast, intraoral radiography showed substantial agreement for symmetry and the presence of CLs, with kappa values between 0.61 and 0.80. Other categories evaluated with intraoral radiography, such as CL shape and periodontal ligament space, showed moderate inter-observer agreement with kappa values between 0.41 and 0.60, whereas lamina dura and cancellous bone pattern showed fair agreement with kappa values below 0.270. Tables 3, 4 present the inter-observer agreement for each morphologic feature evaluated in intraoral radiography and CBCT.

**Table 3:** Inter-observer agreement with IC 95% for the chevron lucency presence and the morphologic features using lateral intraoral radiography in 82 teeth.

Morphologic feature	Agreement (%)	Weighted Cohen’s kappa (*κ*) (Quadratic)	95% CI	Strength of agreement
CL Presence	81.7	0.782	(0.645–0.899)	Substantial
CL Symmetry	82.9	0.658	(0.488–0.805)	Substantial
CL Shape	70.7	0.563	(0.422–0.698)	Moderate
PDL Space	62.2	0.462	(0.328–0.591)	Moderate
Cancellous Bone Pattern	93.9	0.270	(0–0.661)	Fair
Lamina Dura	53.7	0.235	(0.064–0.386)	Fair

PDL, Periodontal ligament; CL, chevron lucency. κ < 0.00 poor; 0.00–0.20 slight; 0.21–0.40 fair; 0.41–0.60 moderate; 0.61–0.80 substantial and 0.81–1.00 almost perfect.

**Table 4:** Inter-observer agreement with IC 95% for the chevron lucency presence and the morphologic features using CBCT in 82 teeth.

Morphologic feature	Agreement (%)	Weighted Cohen’s kappa (*κ*) (Quadratic)	95% CI	Strength of agreement
CL Symmetry	97.6	0.932	(0.820–1.000)	Almost Perfect
CL Shape	87.8	0.812	(0.693–0.905)	Almost Perfect
CL Presence	90.2	0.925	(0.865–0.971)	Almost Perfect
Cortical Bone (near Apex) *	86.6	0.700	(0.518–0.851)	Substantial
PDL Space	73.2	0.621	(0.480–0.739)	Substantial
Lamina Dura	75.6	0.521	(0.330–0.694)	Moderate
Cancellous Bone Pattern	72.0	0.445	(0.149–0.656)	Moderate

PDL, Periodontal ligament; CL, chevron lucency. *This feature corresponds to the cortical bone adjacent to the apex on the nasal and buccal regions; this feature was only evaluated in CBCT. κ < 0.00 poor; 0.00–0.20 slight; 0.21–0.40 fair; 0.41–0.60 moderate; 0.61–0.80 substantial and 0.81–1.00 almost perfect.

Most performance levels for sensitivity and specificity for CL presence were below 70% across the three scores (not visible [0], partially seen [1], and well-defined [2]), except for the specificity of 82.9% for score 1. For CL symmetry, all scores (symmetrical (1) and asymmetrical (2)) for sensitivity and specificity had performance levels below 80%. Similarly, the sensitivity and specificity levels for the lamina dura were moderate, with values under 80%. For the periodontal ligament space, only the score (0) Not visible had a sensitivity above 90%. The performance levels for cancellous bone showed a sensitivity of 98.4% for score 1 (partially seen) and 98.4% for score 2 (fine, uniform trabecular bone), while the rest of the values were very low. Table 5 shows the performance levels for each score in each category.

**Table 5:** Sensitivity and specificity average calculations- one vs. all procedure (intraoral radiography vs. CBCT-reference standard).

Morphologic feature	Score levels	Sensitivity %	Specificity %	PPV %	NPV %	Accuracy %
CL presence	0	63.4	57.2	27.2	86.2	58.6
	1	4.6	82.9	4.2	87.7	74.4
	2	38.1	64.8	70.5	32.1	46.4
CL symmetry	0	73.4	60.4	35.8	88.5	63.5
	1	60.4	73.4	88.5	35.8	63.5
CL shape	0	21.8	81.5	20.2	83.3	71.4
	1	0	91.3	0	89.5	82.4
	2	20.7	70.6	43.7	44.7	44.5
	3	63.4	56.5	26.8	86.1	57.9
Lamina dura	1	64	28.9	49	45.4	45.8
	2	20	72.8	45.7	49.4	45.1
PDL space	0	7.2	95.4	12.5	92.9	89
	1	38.7	52.1	50.4	40	43.9
	2	47.6	44.8	33.2	59.1	45.1
Cancellous bone	0	0	1	-	96.4	96.4
	1	3.4	95.4	8.4	76.6	73.8
	2	98.4	12.6	75.4	83.4	75

Performance levels for sensitivity and specificity: Excellent (>90%), highly reliable; Good (80–90%), suitable for clinical use; Moderate (70–80%), requiring clinical caution; and Poor (<70%), not to be used for critical decision-making in isolation. CL, chevron lucency; PDL, periodontal ligament space; PPV, positive predictive value; NPV, negative predictive value.

**Figure 3:**
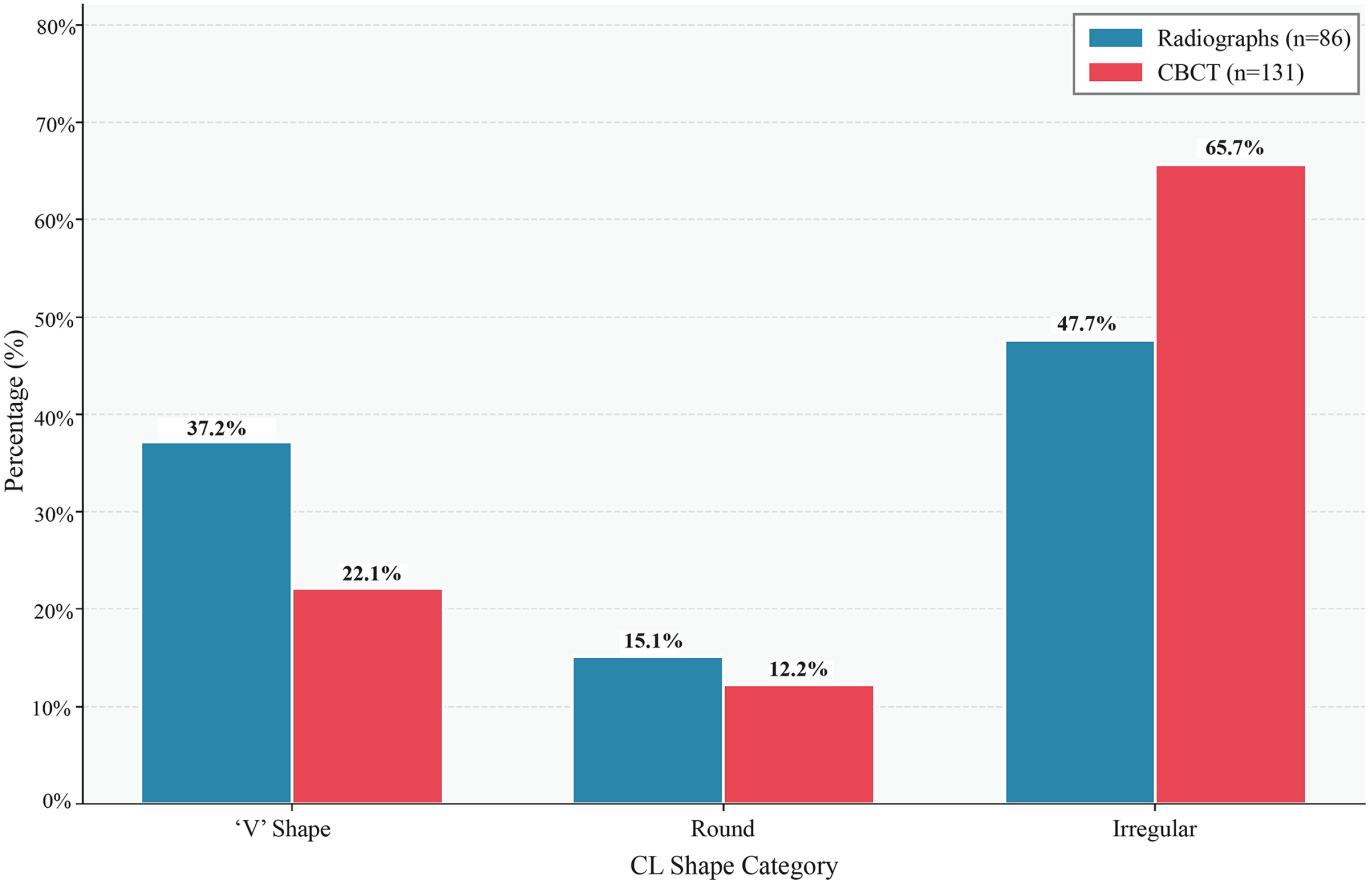
CL shape distribution in radiographs and CBCT. Only visible cases were included (radiography, *n* = 86; CBCT, *n* = 131), using both observers combined. Note that the irregular shape was the most common form observed in both dental radiography and CBCT.

**Figure 4:**
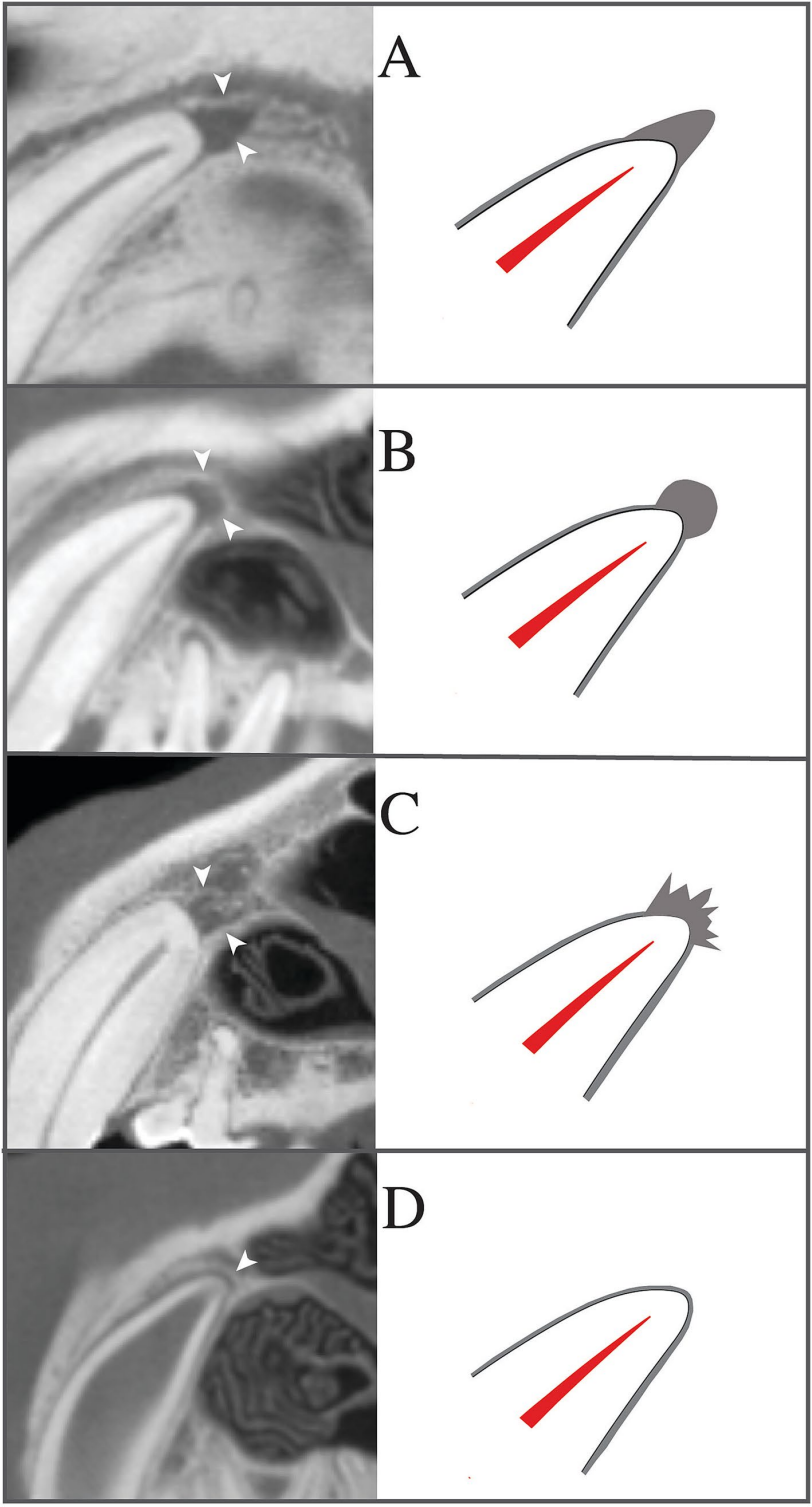
Sagittal CBCT multiplanar reconstructions and corresponding schematic diagrams of canine teeth in adult dogs, illustrating the various morphological forms of chevron lucencies identified in this study (arrowheads): **(A)** V-shaped, **(B)** Round, **(C)** Irregular, and **(D)** Absent.

After identifying almost perfect agreement in CL shape between the two evaluators, observations were pooled to maximize the analytical sample size, and percentages were calculated relative to the total number of CLs present in intraoral radiography (*n* = 86) and CBCT (*n* = 131). Overall, the CL shape distribution for both observers showed that the irregular shape was the most common morphology for both imaging modalities, with 47.7 and 65.7% for intraoral radiography and CBCT, respectively. The second most frequent shape was the ‘V’ shape, with 37.2 and 22.1% for intraoral radiography and CBCT, respectively. Figure 3 displays the consolidated distribution for both observers among visible CLs, and Figure 4 shows its CBCT appearance.

Before fitting the final cumulative link mixed model, a multiple regression was performed to assess the correlation between each covariate and the CBCT CL presence variable. For the variable skull type, due to the sparsity of samples, Fisher’s exact test showed that the association between skull type and CBCT CL presence was not significant in the current dataset, with *p-*values of 0.650 and 0.719 for observers one and two, respectively. Therefore, it was not included in the final model. For other variables, including sex, age, and body weight, the univariate regression tests showed overall *p-*values < 0.2; therefore, we included these variables in the final model.

Brachycephalic dogs had the highest identification rate for CL presence at 100% (*n* = 8/8 observations), followed by dolichocephalic dogs at 87.5% (*n* = 14/16 observations). Mesaticephalic dogs, the largest group in the study, had an identification rate of 77.9% (*n* = 109/140 observations). Overall, CBCT identified the CL in 131 of 164 evaluations conducted by the two evaluators. Body weight and sex were significant predictors of CL identification, with *p*-values of <0.001 and 0.04, respectively. For every 1 kg increase in body weight, the odds of CL detection increased by 17% (OR = 1.17, 95% CI: 1.06–1.28). Males had 91% lower odds of CL identification than females (OR = 0.09, 95% CI: 0.01–0.75), although the wide confidence interval should be interpreted with caution. In contrast, age did not show a statistically significant relationship with CL presence (*p =* 0.113; OR = 0.97, 95% CI: 0.95–1).

The cortical bone structure near the apex (maxillary, nasal, and labial plates) was evaluated solely using CBCT. The most common finding was well-defined, continuous, and uniform, observed in 70.7% (*n* = 58) of the samples. Partially observed, poorly defined was noted in 24.4% (*n* = 20) of the cases, often associated with areas of thinner trabecular density. Lastly, the cortical bone was not visible in only 4.9% (*n* = 4) of the teeth. Multivariable ordinal logistic regression identified body weight as a significant predictor of cortical bone visualization near the apex (*p* < 0.001). Dogs with well-defined cortical plates (score 2) had a higher mean body weight (18.4 ± 6.2 kg) than those with limited visibility or absent cortical plates (scores 0 and 1 combined; 12.1 ± 4.8 kg). Each 1-kg increase in body weight was associated with 22% higher odds of achieving a higher cortical bone visualization score (OR = 1.22, 95% CI: 1.08–1.37). Age and sex were not significant, with *p*-values of 0.315 and 0.10, respectively.

## Discussion

Consistent with previous studies, cone-beam computed tomography (CBCT) proved to be the most reliable imaging modality for identifying nearly all analyzed morphological features, showing better agreement than intraoral radiography and only moderate agreement for the lamina dura and the periapical cancellous bone pattern (16–18). This advantage is mainly due to CBCT’s ability to eliminate structural superimposition, a limitation common to conventional two-dimensional imaging. These 2D limitations can be especially challenging in the maxillary canine region during lateral intraoral radiography acquired with the bisecting technique, where X-ray beam angulation often causes the tooth root to overlap complex maxillofacial structures, such as the conchal crest or the incisivomaxillary canal, as previously reported (19).

Although CBCT showed better overall agreement than intraoral radiography, agreement regarding cancellous bone and the lamina dura was only fair in dental radiography. The low agreement in intraoral radiography likely results from the limitations of assessing a three-dimensional structure in a two-dimensional image. In CBCT, the same variables showed only moderate agreement, which may be due to patient size, especially in small dogs, where the thinness of the cancellous bone trabeculae may be close to the equipment’s voxel size, leading to partial-volume effects and decreased image detail (20). Alternatively, another imaging method, such as micro-computed tomography, could improve visualization of the periradicular trabecular architecture; however, its clinical use remains limited due to high radiation doses, long acquisition times, and a limited field of view (21, 22).

The prevalence of irregular shapes observed in intraoral radiographs may be attributable to variability inherent to the bisecting-angle technique, particularly given that the images were acquired by multiple operators. This variability may have resulted in foreshortening or elongation artifacts, thereby altering the apparent morphology of the apical region. As demonstrated in a previous study, the radiographic appearance of the root apex of the maxillary canine can also be influenced by superimposition with nasal and maxillary structures (19). Future prospective studies incorporating standardized patient positioning and X-ray beam angulation are recommended to mitigate this limitation.

In sagittal CBCT reconstructions, the irregular appearance may be attributed to the ability to visualize trabecular bone without structural superimposition, resulting in a more detailed representation of periapical morphology. Another notable finding was the presence of a round CL conformation. Although rarely observed, this feature should be interpreted in conjunction with clinical findings to rule out true periapical disease. In the present study, this morphology was identified in clinically healthy teeth, in contrast to the existing literature, in which balloon-like periapical lucencies are typically associated with endodontic disease (23). However, the interpretation of this morphology as a normal anatomical variant should be approached with caution, as our retrospective study did not incorporate histopathological assessment of the evaluated teeth, and tooth vitality was determined solely based on clinical examination and radiographic evidence.

When CBCT was used as the reference standard, the diagnostic performance of intraoral radiography varied notably across morphological features. The highest sensitivity was observed for the cancellous bone pattern (98.4%, score 2), indicating that radiography reliably detects well-defined trabecular bone when present. In contrast, the PDL space and lamina dura showed considerably lower sensitivity, ranging from 7.2 to 47.6% and from 20 to 64%, respectively, suggesting that intraoral radiography frequently fails to detect these structures as visualized by CBCT. Similarly, CL presence and symmetry showed moderate sensitivity (ranging from 38.1 to 73.4%), indicating that radiography misses a substantial proportion of these features visible on CBCT, consistent with studies in humans (24).

The low specificity observed for cancellous bone (12.6%, score 2) and PDL space (44.8–52.1%, scores 1 and 2) indicates a pronounced tendency toward false positives for these features, likely attributable to anatomical superimposition inherent to two-dimensional imaging. Notably, the negative predictive value for CL presence (32.1%, score 2) and PDL space (40%, score 1) was markedly low, suggesting that negative radiographic findings cannot reliably exclude these structures. Conversely, cancellous bone absence (score 0) showed a high NPV of 96.4% and near-perfect specificity (100%), reflecting that when cancellous bone is not detected radiographically, it is also unlikely to be visible on CBCT. Collectively, these findings reinforce CBCT as the reference method of choice for definitive periapical morphological assessment, consistent with the existing literature demonstrating the superiority of three-dimensional imaging over conventional radiography (24).

CBCT was used as the reference standard in the diagnostic performance analysis comparing intraoral radiography and CBCT, given its well-established superiority in the visualization of three-dimensional structures over conventional two-dimensional imaging (25). However, histopathological analysis is considered the true gold standard for ruling out pathosis and confirming the normality of the evaluated structures (26). In the present study, histopathology was not feasible due to its retrospective nature and the ethical considerations inherent in sampling tissues from living patients. Future studies on extracted teeth from anatomical specimens could incorporate histopathological analysis, enabling direct correlation with CBCT findings and further validating the imaging criteria described here.

Contrary to what has been described in the literature, where intraoral radiography shows the CL as a V-shaped area circumscribing the dental apex in dogs, our findings indicated that the most common CL shape across both diagnostic imaging methods was irregular (27). This is an interesting finding; however, this result should be interpreted with caution in CBCT because the sagittal reconstruction used in our study did not replicate the projection angle used to acquire the intraoral radiographs. Although this concern was raised during the training session, sagittal reconstruction was chosen for standardization because it produced images comparable to those from oblique reconstructions. Because dorsal and oblique multiplanar reconstructions were not included in the analysis, their potential role in defining CL shape warrants further investigation in future studies.

The lack of significance of skull conformation, age, and sex as predictors of CL detection can be explained by several factors. First, the CL is considered a normal anatomical finding in healthy dogs rather than a pathological lesion, so its presence would be expected across all individuals regardless of their shape or demographics. Second, the limited sample size, especially the underrepresentation of brachycephalic (*n* = 2) and dolichocephalic (*n* = 4) dogs compared to the mesaticephalic group (*n* = 35), may have reduced the statistical power needed to detect meaningful differences between skull conformation groups. Lastly, although age-related changes in dental and periapical structures might be expected, our results suggest that once the tooth is fully erupted and developed, the CL can remain a consistent anatomical feature regardless of the animal’s age.

While age and sex were not statistically linked to CL presence, body weight was a significant factor in CL detection (*p* < 0.001). Although skull conformation consistently showed high identification rates (above 70%) across groups, it did not reach statistical significance in identifying the CL. These results suggest that body weight might have a more important role in CL detection than cranial shape alone. The observed differences in identification rates among brachycephalic, dolichocephalic, and mesaticephalic dogs may be partly due to the larger number of mesaticephalic dogs in the study, which could have affected the overall distribution and reduced the identification rate for this group.

Periapical cortical bone architecture was evaluated solely using CBCT because two-dimensional intraoral radiography is inherently limited by overlapping structures and cannot reliably assess this anatomical feature. CBCT enabled the characterization of normal imaging parameters of the cortical plates near the apex, including the cortical bone surrounding the incisivomaxillary canal. Cortical bone was identified in most cases (95.1%) when combining clearly visible and partially visible categories, while it was not seen in only 4.9% of cases. Disruption of cortical integrity, such as bone lysis or periosteal reaction associated with trabecular destruction in the periapical region, may indicate endodontic disease resulting from pulp necrosis and infection, as previously reported (7, 24). Therefore, accurate detection of these changes is crucial for proper diagnosis and treatment planning.

On the other hand, difficulty visualizing cortical bone in some cases may stem from voxel size. In our study, we used multiplanar reconstructions with an isotropic voxel size of 0.3 mm. When combined with patient-related factors, such as body weight, since smaller dogs tend to have thinner cortical plates, this may have influenced CL detection. As previously mentioned, when cortical bone thickness is close to the voxel size, spatial resolution may be affected by partial volume effects (20). This can result in blurred cortical edges and make thin plates appear discontinuous or missing, even if they are present. Future studies examining other dog tooth types might consider smaller voxel sizes to better visualize cortical detail; however, this could also increase image noise and potentially reduce overall diagnostic quality.

Several limitations of this study stem from its retrospective design, particularly regarding the acquisition of intraoral radiographs. Ideally, intraoral radiography of the maxillary canine includes two standard orthogonal views to minimize overlap of the maxillary bone and to comprehensively assess the dental structures and surrounding alveolar bone. However, due to the retrospective nature of this research, diagnostic imaging protocols were not standardized, and occlusal views were not consistently available. Consequently, radiographs were obtained by multiple operators, and only lateral views obtained with the bisecting-angle technique could be evaluated.

Because these lateral images were taken by different staff members, projection angles may have varied slightly among operators. This lack of standardization is particularly significant when evaluating periapical regions, as even minor variations in projection angles can alter visualization, reduce the perceived clarity of periapical structures, and lead to inconsistent interpretations (19). Furthermore, this geometric variability severely compromises the reliability of using radiographic symmetry to distinguish normal from pathological findings. While clinicians in a practice setting frequently compare right and left radiographs of the same patient to identify abnormalities, subtle variations in beam angulation between sides can artificially create, exaggerate, or mask periapical lucencies. Therefore, relying strictly on bilateral radiographic symmetry as a diagnostic baseline is highly problematic unless positioning is perfectly identical and standardized.

While future prospective studies should implement standardized imaging protocols and patient positioning to ensure a complete, bilateral, orthogonal assessment, it is worth noting that strict standardization may not fully reflect a real-world clinical setting. In routine practice, radiographs are commonly obtained by multiple staff members with inherent inter-operator variability, making a thorough understanding of these geometric limitations essential for accurate diagnostic interpretation.

Another limitation of our study was the small number of brachycephalic and dolichocephalic dogs in the sample population. The overrepresentation of mesaticephalic dogs prevented us from accurately identifying the most common lucency shape across different skull conformations. Future studies with more brachycephalic and dolichocephalic dogs would help produce clearer results.

A final limitation to acknowledge is that our study evaluated teeth classified as clinically healthy based solely on clinical and imaging findings. Because pulp vitality testing and histopathological evaluations were not performed, additional diagnostic criteria were unavailable. This is particularly relevant in cases where early widening of the periodontal ligament space or the presence of round or irregular periapical lucencies might also be associated with early endodontic pathosis. Consequently, further studies incorporating endodontically affected teeth are necessary to determine whether the diagnostic imaging features identified in the current study are sufficient to accurately differentiate normal anatomical variations from active endodontic disease.

## Conclusion

The findings of this study support rejecting the null hypothesis, confirming that CBCT provides greater diagnostic reliability than intraoral radiography in characterizing chevron lucency and periapical structures in the maxillary canine teeth of dogs. The irregular shape was the most common CL morphology across both methods, and body weight and sex were the primary predictors for CL presence. While intraoral radiography remains a useful screening tool, CBCT is recommended for definitive periapical evaluation when radiographic results are inconclusive or detailed morphological information is needed for clinical decisions.

## Data Availability

The raw data supporting the conclusions of this article will be made available by the authors, without undue reservation.

## References

[1] Hermanson J De Lahunta A Evans H. "The skeleton". In: Hermanson J, De Lahunta A, Evans H, editors. Miller and Evans’ Anatomy of the dog, 5th Edn. St. Louis, MO: Elsevier (2020). p. 86–116.

[2] Fiorellini J Stathopoulou P. Anatomy of the periodontium. In: Newman MG Takei H Klokkevold PR Carranza FA, editors. Carranza’s clinical periodontology, 12th ed St. Louis, MO: Elsevier Saunders (2015) 9–39.

[3] DuPont GA DeBowes LJ. "Intraoral radiographic anatomy of the dog". In: DuPont GA, DeBowes LJ, editors. Atlas of dental Radiography in Dogs and cats, 1st Edn. Philadelphia, PA: Saunders Elsevier (2009). p. 5–80.

[4] White S Pharoah M. "Normal radiographic anatomy". In: Oral Radiology Principles and Interpretation, 6th Edn. St. Louis, MO: Mosby Elsevier (2009). p. 152–74.

[5] Fiani N Arzi B Pritchard WB. Diagnostic imaging in veterinary dental practice. J Am Vet Med Assoc. (2010) 236:41–3. doi: 10.2460/javma.236.1.41, 20043796

[6] Jorge EG Tanomaru-Filho M Gonçalves M Tanomaru JMG. Detection of periapical lesion development by conventional radiography or computed tomography. Oral Surg Oral Med Oral Pathol Oral Radiol Endod. (2008) 106:e56–61. doi: 10.1016/j.tripleo.2008.03.020, 18585613

[7] Karamifar K Tondari A Saghiri MA. Endodontic periapical lesion: an overview on the etiology, diagnosis and current treatment modalities. Eur Endod. (2020) 5:54–67. doi: 10.14744/eej.2020.42714, 32766513 PMC7398993

[8] Siqueira JF Silva WO Romeiro K Gominho LF Alves FRF Rôças IN. Apical root canal microbiome associated with primary and posttreatment apical periodontitis: a systematic review. Int Endod J. (2024) 57:1043–58. doi: 10.1111/iej.14071, 38634795

[9] Huumonen S Ørstavik D. Radiological aspects of apical periodontitis. Endod Top. (2002) 1:3–25. doi: 10.1034/j.1601-1546.2002.10102

[10] de Paula-Silva FWG Júnior MS Leonardo MR Consolaro A da Silva LAB. Cone-beam computerized tomographic, radiographic, and histologic evaluation of periapical repair in dogs’ post-endodontic treatment. Oral Surg Oral Med Oral Pathol Oral Radiol Endod. (2009) 108:796–805. doi: 10.1016/j.tripleo.2009.06.016, 19734073

[11] Mohan R Singh A Gundappa M. Three-dimensional imaging in periodontal diagnosis - utilization of cone beam computed tomography. J Indian Soc Periodontol. (2011) 15:11–7. doi: 10.4103/0972-124X.82256, 21772715 PMC3134038

[12] Campbell RD Peralta S Fiani N Scrivani PV. Comparing intraoral radiography and computed tomography for detecting radiographic signs of periodontitis and endodontic disease in dogs: an agreement study. Front Vet Sci. (2016) 3:31. doi: 10.3389/fvets.2016.00068, 27630993 PMC5005414

[13] Geibel MA Schreiber ES Bracher AK Hell E Ulrici J Sailer LK . Assessment of apical periodontitis by MRI: a feasibility study. RoFo. (2015) 187:269–75. doi: 10.1055/s-0034-1385808, 25594373

[14] Gamer M Lemon J Fellows I Singh P. irr: Various Coefficients of Interrater Reliability and Agreement. R package version 0.84. Available at CRAN Package irr (2012).

[15] Hicks SA Strümke I Thambawita V Hammou M Riegler MA Halvorsen P . On evaluation metrics for medical applications of artificial intelligence. Sci Rep. (2022) 12:5979. doi: 10.1038/s41598-022-09954-8, 35395867 PMC8993826

[16] Emilio S De Sousa M Pena L de Sousa SEM Monteiro LPB. Diagnostic accuracy of cone-beam computed tomography and periapical radiography in detecting external cervical resorption: a systematic review and meta-analysis. Aust Endod J. (2026) 52:272–81. doi: 10.1111/aej.70035, 41273117

[17] Piecha MCR Pappen FG Gomes F d A Sfreddo CS Pola NM. Cone beam computed tomography vs. periapical radiograph: diagnostic accuracy in endo and periodontal lesions. Braz Dent J. (2026) 36:e256704. doi: 10.1590/0103-644020256704, 41637255 PMC12872107

[18] Sheikhi M Abdinian M Roshanzamir N Aghaziarati F. Comparison of periapical parallel radiography with cbct with different field of views (FOV) for the detection of periapical lesions. Dent Res J (Isfahan). (2024) 21:67. doi: 10.4103/drj.drj_466_23, 39802812 PMC11722740

[19] Gracis M Harvey CE. Radiographic study of the maxillary canine tooth in mesaticephalic dogs. J Vet Dent. (1998) 15:73–8. 10597154 10.1177/089875649801500202

[20] Brüllmann D Schulze RKW. Spatial resolution in CBCT machines for dental/maxillofacial applications - what do we know today? Dentomaxillofac Radiol. (2014) 44:20140204. doi: 10.1259/dmfr.20140204, 25168812 PMC4614158

[21] Morin MC D’Astous J. A micro-CT study of the pulp cavity morphology of maxillary fourth premolar teeth in dogs. Front Vet Sci. (2024) 11:1499465. doi: 10.3389/fvets.2024.1499465, 39620112 PMC11605712

[22] Calvo-Guirado JL Carlos-Villafranca F De Garcés-Villalá M García-Carrillo N Jindal V Martínez-Martinez F X-ray micro-computed tomography characterization of autologous teeth particle used in post extraction sites for bone regeneration. An experimental study in dogs Indian J Dent Sci (2022) 2 58–67 doi: 10.4103/ijds.ijds_138_21

[23] Zanini M Decerle N Hennequin M Cousson PY. Revisiting Orstavik’s PAI score to produce a reliable and reproducible assessment of the outcomes of endodontic treatments in routine practice. Eur J Dent Educ. (2021) 25:291–8. doi: 10.1111/eje.12603, 32966674

[24] Estrela C Bueno MR Azevedo BC Azevedo JR Pécora JD. A new periapical index based on cone beam computed tomography. J Endodont. (2008) 34:1325–31. doi: 10.1016/j.joen.2008.08.013, 18928840

[25] Lo Giudice R Nicita F Puleio F Alibrandi A Cervino G Lizio AS . Accuracy of periapical radiography and CBCT in endodontic evaluation. Int J Dent. (2018) 2018:2514243. doi: 10.1155/2018/2514243, 30410540 PMC6206562

[26] Rosenberg PA Frisbie J Lee J Lee K Frommer H Kottal S . Evaluation of pathologists (histopathology) and radiologists (cone beam computed tomography) differentiating radicular cysts from granulomas. J Endodont. (2010) 36:423–8. doi: 10.1016/j.joen.2009.11.005, 20171356

[27] DuPont GA DeBowes LJ, (editors). "Endodontic disease". In: Atlas of dental Radiography in Dogs and cats, 1st Edn. Philadelphia, PA: Saunders Elsevier (2009). p. 142–71.

