# Correction: Radiographic and cone-beam computed tomography characterization of Chevron lucency in 82 clinically healthy maxillary canine teeth of dogs: a retrospective descriptive study

**DOI:** 10.3389/fvets.2026.1932071

**Published:** 2026-07-28

**Authors:** Lenin A. Villamizar-Martinez, Yang Qu, Cristian M. Villegas Lobos, Fernanda M. Lopes-Kubitza

**Affiliations:** 1 Dentistry and Oral Surgery Service, Department of Clinical Science, North Carolina State University, Raleigh, NC, United States; 2 Clinical Studies Core, Office of Research, College of Veterinary Medicine, North Carolina State University, Raleigh, NC, United States; 3 Luiz de Queiroz College of Agriculture, São Paulo University, Piracicaba, SP, Brazil

**Keywords:** Chevron lucency, cone beam tomography, diagnostic imaging, dogs, intraoral radiography, periapical lucency, tooth apex

Author Lenin A. Villamizar-Martinez was erroneously included as corresponding author. The correct corresponding author is Fernanda M. Lopes-Kubitza.

The original version of this article has been updated.

